# Evidence-based and experience-based medicine, which comes first: the chicken or the egg?

**DOI:** 10.52054/FVVO.15.3.090

**Published:** 2023-09-24

**Authors:** B Ata, E Saridogan

**Affiliations:** Koc University, Department of Obstetrics and Gynecology, Istanbul, Turkey; ART Fertility Clinics, Dubai, United Arab Emirates; University College London, Elizabeth Garrett Anderson Institute for Women’s Health, London, United Kingdom; University College London Hospital, Women’s Health Division, London, United Kingdom

Guidelines have become indispensable tools for clinical practice. Guidelines prepared by international or national professional societies, e.g., European Society for Gynaecological Endoscopy (ESGE), European Society of Human Reproduction and Embryology (ESHRE) and American Society of Reproductive Medicine (ASRM), or national authorities, e.g., National Institute for Health and Care Excellence or governmental bodies do not only influence decision making in the clinic but can also form the basis for reimbursement (or lack of it) of diagnostic and therapeutic procedures.

The majority of the above-mentioned guidelines are evidence-based and follow a well-defined methodology for preparation. The steps for evidence-based guideline development include the recruitment of content experts from diverse backgrounds related to the clinical condition of interest as well as methodologists and users including patients. The guideline development group formulates questions to be answered, which often follow a certain format such as PICO (population, intervention, comparison, outcome) as much as possible (Vermeulen et al., 2019). Once the questions are prepared, an extensive systematic literature search is undertaken. While this sounds relatively straightforward with currently available electronic resources, selection, and categorisation of relevant publications from a usually overwhelming number of identified citations followed by extraction of relevant information and data from the selected publications are arduous tasks requiring vast resources in terms of time and effort. Collating results from different publications, reconciling their differences to come up with actionable recommendations for clinical practice often takes days of long discussions. The vast majority of current guidelines follow the well-established GRADE approach at this stage (Guyatt et al., 2008). However, despite the presence of this structured method, some subjectivity in interpretation remains inevitable. Thus, different evidence-based guidelines prepared by different groups, or different editions of the same guidelines on one subject can recommend different approaches, albeit rarely. These variations are not necessarily brought about by availability of conflicting studies (read as evidence) for different guidelines but at times by different interpretation of the “quality” of available evidence, or prioritisation of different values and preferences. The lack of “high quality” or even acceptable quality evidence more often than not prevents formulation of an evidence-based recommendation, and many guidelines contain recommendations based on expert opinions (read as experience) of the guideline development groups. Often, the final drafts of guidelines are opened for feedback from stake holders, e.g., members of the professional society authoring the guideline, patients, ethicists as well as legal professionals before the final version is presented for use. Despite all the efforts vested by ideally a diverse and inclusive guideline development group, rarely all the recommendations are formulated unanimously, or they are well accepted by all stake holders. Since, medical knowledge develops at a rapid pace, clinical practice guidelines require updating after a period, which may or may not be predefined. All the above-mentioned steps need to be repeated for an update, consuming similarly substantial effort, time, and funds as the former edition.

In this issue of Facts, Views and Vision we publish two articles that are relevant to the methodology of evidence-based guidelines and their place in guiding the clinical practice. Tummers et al. (2023) present an analysis of changes in the recommendations in the update of the Dutch Minimally Invasive Surgery Guideline after 10 years from its first version. The first point that catches attention is the update being delayed by five years from the original intended date of update, due to the non-availability of necessary resources. It is noteworthy and understandable that only one of the members of the guideline development group had participated in the development of both editions. The new guideline development group did not consider 15 of the 26 key issues identified in the 2011 edition relevant for updating and introduced two new key issues. The 13 key questions resulted in 15 recommendations. Only two of the recommendations remained the same as before. Nine (60%) recommendations were changed despite the evidence-based conclusions remaining unchanged, and only two (13%) recommendations were changed due to a change in evidence-based conclusions. While the majority of the recommendations differed between the two editions, it seems that it was the interpretation of the evidence that led to the changes rather than change in evidence base. While this can be regarded as an example of the inevitable subjectivity in interpretation of evidence, it can also be caused by increased cumulative clinical experience of the guideline developers over the ten-year period, even in the absence of new high-quality evidence (which excludes expert opinion by definition) to inform these recommendations. On a further note, the authors suggest that guideline development groups should identify issues that may not need updating in future editions to save time and effort in the process. It remains to be seen how accurate the guideline development group prediction is in determining the likelihood of new evidence in the future and whether this approach may reduce the strength of guideline methodology.

The other article in this issue by Wattiez et al. (2023) describe their attempt to structure ‘experience’ into what they call ‘experience-based medicine’. While recognising the value of evidence-based medicine, the authors highlight the difficulty of generating “high quality” evidence to guide the management of patients with endometriosis, which is a multi-faceted complex condition that has various presentations during different stages of life. Endometriosis can cause diverse complaints and symptoms, depending on location and extent of the disease, besides other unknown factors. Multiple management options, ranging from various medical treatments to a multitude of surgical methods exist. Assisted reproductive technology is among the options for patients suffering from endometriosis related infertility. Naturally these options come with different effectiveness, side effects and risk profiles as well as at different costs. The most appropriate choice is highly individualised depending on numerous factors including age, patients’ priorities and preferences, accessibility of treatment as well as experience of medical professionals. It is probably an elusive goal to design and execute numerous randomised controlled trials with adequate sample size and proper design features that would provide high quality evidence applicable to each and every individual with endometriosis in endless different clinical settings. While multiple evidence-based guidelines are available for the management of endometriosis, yet many recommendations are based on low quality evidence in the form of poor-quality studies or merely expert opinions. Experience-based medicine is an approach to medical practice that emphasises the use of both clinical expertise and patient experiences in making clinical decisions. It recognises that medical decisions should be based not only on scientific evidence but also on the practitioner’s experience and the patient’s unique circumstances and preferences. Experience based medicine seeks to integrate the best available evidence from research with clinical judgment to provide the most appropriate care for each individual patient. It values the input of both healthcare providers and patients in the decision-making process, aiming to improve the overall quality of care. However, experience is individual in essence, and as such it is subject to many biases from a strict scientific point of view. A major concern for experience-based decision making is its reproducibility. And as such experience in the form of “expert opinion” is regarded to provide low quality evidence. Wattiez et al. (2023) address this issue by analysing responses to questionnaires by a diverse group of experts with different backgrounds and experience levels. They make an attempt to differentiate decisions based on experience or evidence from the literature and concur that the “collective experience-based conclusions” are in good agreement with the recommendations from the ESHRE evidence-based endometriosis guideline (Becker et al., 2022). It seems if multiple experts are of the same opinion, it is more likely to be correct, and perhaps should not be looked down on as the lowest quality evidence by default. Wattiez et al. (2023) acknowledge the difficulty of quantifying to what extent the “experience-based conclusions” were already informed by the “evidence”.

Both papers are interesting to read and provide food for thought. In our opinion they both highlight the fact that evidence-based and experience-based medicine have their own limitations and are not mutually exclusive but rather complementary for sound clinical practice. Indeed, evidence-based medicine, by definition, integrates the best available scientific evidence with clinical expertise and patient value and preferences (Sackett et al., 1996). While a formal definition of experience-based medicine is lacking, the essence of the concept is the same as evidence-based medicine; clinical experience, available evidence and patients’ input should direct clinical practice. Perhaps the difference is in the weight given to experience and evidence, or it is just a matter of semantics.
